# Editorial: Epigenetic therapy against cancer: toward new molecular targets and technologies

**DOI:** 10.3389/fcell.2023.1218986

**Published:** 2023-05-24

**Authors:** Ângela Sousa, Christiane P. Soares, Chung Man Chin, Daniela Trisciuoglio, Fatima Valdes-Mora

**Affiliations:** ^1^ CICS-UBI—Health Science Research Centre, University of Beira Interior, Covilhã, Portugal; ^2^ School of Pharmaceutical Sciences, São Paulo State University (UNESP), Araraquara, Brazil; ^3^ School of Medicine, UNION of the Colleges of the GREAT LAKES (UNILAGO), Sao José do Rio Preto, Brazil; ^4^ IBPM Institute of Molecular Biology and Pathology, CNR National Research Council, C/o Department of Biology and Biotechnology “Charles Darwin”, Sapienza University of Rome, Rome, Italy; ^5^ Children’s Cancer Institute Australia Randwick, Randwick, NSW, Australia; ^6^ Garvan Institute of Medical Research Darlinghurst, Darlinghurst, NSW, Australia

**Keywords:** cancer therapy, epigenetics, histone deacetylases, inhibitors, prognostic biomarker, tumor microenvironment

Epigenetics is defined as a group of inheritable changes in gene expression without modifications to the DNA sequence. DNA methylation, histone deacetylation and non-coding RNA expression are examples of epigenetic control. Disruption of the epigenetic program of gene expression is a hallmark of cancer that initiates and propagates tumorigenesis. Considering that epigenetic modifications are reversible, the ability to restore the cancer epigenome through the inhibition of the epigenetic modifiers is a promising therapy for cancer treatment, by monotherapy or in combination with other anticancer therapies, including immunotherapies. Costa et al., summarized the main epigenetic alterations, their potential as a biomarker for early diagnosis and the epigenetic therapies approved for cancer treatment.

Anti-programmed cell death protein 1 (PD-1) or PD-ligand 1 (PD-L1) immune checkpoint therapy has shown exciting clinical outcomes in diverse human cancers. Shao et al., highlighted biological characteristics of exosome PD-L1 in tumor immunity, exosome PD-L1 detection methods and proposed that exosome PD-L1 can be a target for overcoming anti-PD-1/PD-L1 antibody treatment resistance. On the other hand, Jia and Wan, investigated the role and function of inositol-3-phosphate synthase 1 (ISYNA1) in pan-cancer, especially in colon adenocarcinoma (COAD). These authors found that ISYNA1 can be a potential prognostic biomarker in COAD, being positively correlated with the immunosuppressive tumor microenvironment (TME). In breast cancer (BC), the most common tumor in women, Zou et al., analyzed the expression, alteration, prognosis, and biological functions of various KCNKs genes. They verified that seven KCNKs genes can regulate breast cancer progression via modulating immune response and established a specific prognostic signature using these genes as ideal biomarkers for breast cancer patients. Xiong et al., observed that DNA-methylation-based biomarker for body mass index (DM-BMI)-related genes were mostly involved in the process of cancer immunity, being positively correlated with immune checkpoint inhibitors (ICI) response markers in BC. In addition, Liu et al., found that NPY5R was frequently downregulated in BC tissues, due to its aberrant promoter CpG methylation, being considered a candidate biomarker. In consequence, ectopic expression of NPY5R significantly reduced breast tumor cell growth, induced cell apoptosis and G2/M arrest, acting as a tumor suppressor. Moreover, NPY5R also promoted the sensitivity of BC cells to chemotherapy by doxorubicin.

Multiple myeloma (MM) is an incurable clonal plasma cell malignancy, being fundamental its understanding and search for efficient therapeutic options. Vlummens et al., identified protein arginine methyltransferase 5 (PRMT5) as a promising prognostic target involved in DNA repair and epigenetics associated with high-risk myeloma, by using bioinformatic tools. They also verified that EPZ015938 strongly reduced the total symmetric-dimethyl arginine levels (PRMT5-inhibitor) in several human myeloma cell lines, leading to a decreased cellular growth and at later time points, apoptosis occurred. Mohammed et al., found that both H3K27 histone demethylases, namely, KDM6A/B, were highly expressed in epithelial cancer cells that lose attachment from the extracellular matrix (ECM) and their inhibition resulted in reduced sphere formation capacity and increased apoptosis.

Glioma is the most common and aggressive malignancy of the central nervous system. Li et al., identified deacetylases (HDACs) 1/2/3/4/5/7/9/10/11 as useful biomarkers for predicting the survival of patients with glioma. Furthermore, HDACs are considered putative precision therapy targets, since their expression are correlated with of immune cell infiltration in patients with glioma. He et al., studied the prognostic value and therapeutic perspectives of CXC chemokine receptor (CXCR) members, a complex of the immune-associated protein involved in tumor activation, invasion, migration, and angiogenesis through various chemical signals, in the glioma microenvironment. Zheng et al., identified a new n6-methyladenosine (m6A) methylation modification patterns and TME infiltration landscape that predict clinical outcomes for papillary renal cell carcinoma patients. In parallel, Luo et al., evaluated and characterized m6A regulator-mediated methylation modification patterns and verified that m6A modification plays an essential role in TME infiltration in ovarian cancer patients which can guide immunotherapy strategies with ICI. Moreover, Dai and Ye, developed and validated a novel histone acetylation-based gene signature that has a well predictive effect on the prognosis of ovarian cancer and can potentially be applied for clinical treatments.

Colorectal cancer can originate by a dysbiosis configuration, that results in the biofilm formation, production of toxic metabolites, DNA damage in intestinal epithelial cells through the secretion of genotoxins, and epigenetic regulation of oncogenes. Schemczssen-Graeff and Pileggi, highlighted the study of potential bacteria as a complement for cancer treatment, being considered the next-generation probiotics and live biotherapeutic products, can have a controlling action in epigenetic processes, changes in the regulation of genes of microbiome and host.

